# The moral economy of worth: Why rights-based mental health must engage cultural logics of contribution

**DOI:** 10.1017/gmh.2026.10255

**Published:** 2026-06-16

**Authors:** Yasuhiro Kotera

**Affiliations:** 1 University of Nottingham, UK; 2 University of Osaka, Japan; 3 Azerbaijan University, Azerbaijan

**Keywords:** rights-based care, moral economy, contribution, collectivism, long-term orientation

## Abstract

Global mental health advances its commitment to rights-based care grounded in dignity, autonomy and freedom from coercion, assuming worth is inherent and support is a right. This Perspective aims to strengthen that agenda by adding a cultural-implementation lens: in many settings, everyday moral reasoning links worth to contribution (e.g., labour, perseverance and fulfilment of social obligations), creating a moral economy of worth in which legitimacy is earned, relational and conditional. Drawing on cross-cultural psychology, the article argues that contribution-based moralities are especially salient where three cultural dimensions co-occur: restraint, collectivism and long-term orientation. In such contexts, support can be morally misread as unearned reward, rest as indulgence and reduced functioning as moral failure, producing ethical dissonance that shapes help-seeking, clinical practice and policy uptake without implying rejection of human rights. Using Japan as an illustrative case, the article shows how norms of perseverance (ganbaru) and regulated dependence (amae) can intensify contingent self-worth, self-stigma, rapid-return expectations and family hesitation to seek support. To improve legitimacy and sustainability, five strategies are proposed: broaden what counts as contribution; frame recovery as stewardship for future participation; avoid morally triggering eligibility language; use culturally credible lived-experience narratives; and engage institutions across levels that shape moral judgement.

## 抽象的な

世界のメンタルヘルス分野では、人権に基づくケア(尊厳、自律、法的保護、強制からの自由)への取り組みが強まっている。こうした枠組みは「人の価値は生まれながらに備わっており、支援は功績への報酬ではなく権利である」という前提に立つ。しかし多くの文化圏では、価値や正当性は「貢献」によって測られやすい。すなわち、勤勉さ、忍耐、家族や組織における役割遂行、他者に迷惑をかけないことが道徳的規範として重視され、支援の受給や休養は「甘え」「不当な報酬」と誤解され得る。本稿は、人権に基づく改革を弱めるのではなく、その実装可能性を高めるために、文化的な「価値の道徳経済(moral economy of worth)」というレンズを提示する。特に、抑制(Restraint)、集団主義(Collectivism)、長期志向(Long-Term Orientation)が重なりやすい社会では、貢献に基づく道徳が強まり、支援の利用が羞恥や自己烙印化を伴いやすいことを論じる。日本を例に、「頑張る」規範や依存に対する規制が、早期復職圧力や家族の支援回避に結びつく可能性を示す。最後に、①貢献概念の拡張(ケアや自己管理を含む)、②回復を将来の参加を支える責任ある自己管理として位置づける、③道徳的反発を招く制度用語・適格基準の回避、④当事者の語りを用いた規範の再解釈、⑤家族・職場・制度など複数レベルの組織への働きかけ、という5つの方策を提案する。.

## Impact statement

This article offers a practical way to strengthen rights-based mental health reform by introducing a cultural-implementation lens: the “moral economy of worth.” It explains that, in many settings, people judge legitimacy and deservingness through everyday expectations of contribution, such as working, persevering, fulfilling roles and not burdening others. Where these norms are strong (often in cultures characterised by restraint, collectivism and long-term orientation), rights-based messages such as “support is a right” can be morally misread as “unearned reward,” and rest or reduced functioning can be interpreted as irresponsibility. The article synthesises these dynamics and provides five actionable strategies to make rights-based care morally legible: broaden what counts as contribution (including caregiving and self-management), frame recovery as responsible stewardship for future participation, design eligibility language that avoids moral “red flags,” use culturally credible lived-experience narratives to renegotiate norms, and engage institutions across levels (families, workplaces, welfare systems and media) that shape moral judgement.

This matters because expanding services and legal protections alone may not increase uptake if people fear shame, loss of standing or being seen as “not contributing.” By addressing the moral meaning of support, rights-based reform can reduce stigma-driven avoidance, improve engagement and become more sustainable across diverse cultural contexts.

Beneficiaries include people experiencing mental health difficulties and their families (through lower shame and easier help-seeking), clinicians and service leaders (through improved communication and engagement) and policymakers and implementers (through better-designed programmes that achieve inclusion and participation without unintentionally activating stigma).

Global mental health has entered a period of renewed commitment to rights-based care, driven by international frameworks that foreground dignity, autonomy, legal protection and freedom from coercion (Puras and Gooding, 2019; World Health Organization, 2021; Mahdanian et al., 2023). In practice, a rights-based approach in mental health prioritises non-discriminatory access, person-centred and recovery-oriented support in the community, continuity of care and safeguards against coercion and degrading treatment, with service quality judged not only by clinical outcomes but also by dignity, participation and autonomy (World Health Organization, 2021; United Nations, 2022). These initiatives, from the WHO’s guidance on community-based mental health services to the UN’s Convention on the Rights of Persons with Disabilities (United Nations, 2022), share a core ethical assumption: human worth is inherent, not earned. Support, in this vision, is not a reward for merit but a right of all people as human beings. Operationally, this aims to reduce inequalities by ensuring access and continuity are based on need and rights, not perceived deservingness. This Perspective is intended to strengthen the rights-based agenda by identifying a cultural-implementation lens that can improve legitimacy, uptake and sustainability across settings. At the same time, international human rights frameworks have also been critiqued as culturally situated, reflecting particular moral and political traditions (Mutua, 2008; Summerfield, 2012). This strengthens our argument: the dissonance described here can be understood as tension between competing cultural frameworks, reinforcing the need for cultural engagement in implementation.

Yet across many cultural contexts, including but not limited to East Asia, moral understandings of worth do not begin from unconditional dignity. Instead, deeply rooted cultural logics define a person’s value through contribution, especially through labour, perseverance and fulfilling social obligations (Gobel and Miyamoto, 2023). For example, Confucianism defines personhood and moral worth through the fulfilment of social roles, diligent effort and contribution to the collective (Chen, 2023). Confucian ethics stress that personhood is achieved by enacting virtues, such as perseverance, diligence and self-cultivation, within social relationships and roles (Yuan et al., 2023). These expectations form a moral economy of worth: drawing on “moral economy” as an established analytical concept for the normative rules and moral evaluations that organise distributions of recourses and recognition (Thompson, 1971; Fassin, 2009). Here, it refers to a widely shared cultural belief that resources, respect and even moral standing should be distributed in proportion to one’s contribution to family, community or society. Such systems are not simply attitudes: they are moral grammars that organise social life, identity and interpersonal obligations.

This moral economy is captured succinctly in the Japanese proverb “働かざる者食うべからず (Those who do not work shall not eat).” Although often invoked in contemporary Japan, similar notions appear across cultures shaped by Confucian (Chan, 2011), Protestant (Weber, 2001), socialist (van Oorschot, 2000) and even familial duty-based ethics (Doi, 2002). These ideas do not represent resistance to human rights per se. Rather, they represent a different moral ontology of what it means to deserve support, one in which worth is conditional, relational and earned. Global mental health has largely overlooked these cultural moral systems. While global mental health frameworks increasingly emphasise rights-based, person-centred care (e.g., WHO guidance on community mental health services and QualityRights), they rarely make explicit how culturally patterned moral logics of deservingness, such as contribution-based worth, can shape the perceived legitimacy of support and thereby influence uptake and implementation (World Health Organization, 2021, 2025; United Nations, 2022). This article focuses on that under-addressed dimension. As a result, rights-based approaches may confront silent but powerful friction: not necessarily because communities reject rights, but because alternative moral frameworks can shape how value, responsibility and entitlement are understood.

## Cultural configurations behind contribution-based worth

Why do some societies maintain strong contribution-based moralities, while others more readily accept unconditional support as a right? Insights from cross-cultural psychology offer a useful analytical lens (Kirmayer, 2025). Drawing on cross-cultural psychology, this article uses a hypothesis-generating heuristic. Contribution-based worth may be especially salient where three cultural tendencies co-occur: Restraint, Collectivism and Long-term orientation (Hofstede et al., 2010). This triad is presented as a proposition to guide future empirical testing rather than as a settled empirical model, informed by cross-cultural theory and by observed convergences in settings where contribution-based moralities appear salient (e.g., Japan). Hofstede’s national cultural dimensions have been widely used but also extensively critiqued, including concerns about within-country heterogeneity, temporal change and reifying “national culture” (McSweeney, 2002). These dimensions can be used as a pragmatic heuristic to characterise broad cultural tendencies relevant to the argument, not as precise or deterministic measures of individuals or societies.

### *Restraint (*versus *indulgence)*

In high-restraint cultures, such as Japan, South Korea, China, Germany and Romania, people are socialised to prioritise duty over personal desire, self-control over pleasure and moral discipline over self-expression (Hofstede et al., 2010). In such contexts, benefits not earned through effort may be perceived as indulgent, morally questionable or socially destabilising (Felix et al., 2002; Dumbravå, 2017; Chen et al., 2024; Takamatsu et al., 2024). The idea that one could receive support independent of contribution conflicts with a deeply embodied ethic of self-denial and moral discipline. This moral backdrop reinforces the belief that worth must be demonstrated through effort.

### *Collectivism (*versus *individualism)*

Collectivist societies emphasise interconnectedness, role fulfilment and obligations to family and community (Hofstede et al., 2010). A person’s value is not purely individual but relational: one “earns” worth by fulfilling expected roles. Someone unable to contribute due to illness, disability or unemployment may be perceived (and may perceive themselves) as a burden (Fan et al., 2023). In these cultural logics, receiving support without contribution violates the relational ethic that binds groups together. Collectivist logics can also motivate strong obligations to care for vulnerable in-group members. The argument here is therefore not that collectivism universally reduces care, but that where worth is tightly linked to contribution, people may still receive support even as the experience heightened shame and self-stigma, and may delay help-seeking or seek care only when impairment becomes undeniable. In such contexts, the friction may operate more through the conditions and meanings of care (e.g., moralised dependence and pressure for rapid return) than through outright absence of care. Rights-based approaches, which emphasise individual entitlements independent of social roles, thus encounter conceptual resistance.

### *Long-term orientation (*versus *short-term orientation)*

Long-term oriented cultures value perseverance, future-oriented sacrifice and sustained commitment to collective goals (Hofstede et al., 2010). This dimension strengthens expectations of industriousness and social responsibility (Halkos and Skouloudis, 2017; Beugelsdijk and Welzel, 2018). Idleness is not merely impractical; it is morally suspect. In such contexts, “taking time off” for mental health may be viewed not as rest, but as moral failure or irresponsibility (Kotera et al., 2022).

Countries where these three dimensions overlap, such as Japan, China, South Korea, Taiwan, Singapore, Hong Kong, parts of India and certain European societies with strong duty ethics (e.g., Germany and Romania), tend to maintain powerful contribution-based moralities. In these contexts, moral worth is strongly indexed to doing one’s part: reliably fulfilling relational and institutional obligations, exercising self-control over personal desire and sustaining long-term commitments to family, organisation and society (Miller and Bersoff, 1992; Beugelsdijk and Welzel, 2018).

## Japan as an illustrative, not exceptional, case

Japan exemplifies the triadic configuration of restraint, collectivism and long-term orientation. The ethic of ganbaru (頑張る; a moralised norm of persevering and “pushing through” despite difficulty) and the ambivalent social evaluation of amae (甘え; culturally recognisable dependence on others’ indulgence, acceptable in some close relationships but often criticised in public as immature or burdensome) can reinforce conditional worth, where esteem is closely tied to meeting duties and maintaining self-control (Mattig, 2013). Mental health challenges often lead to profound self-stigma, as individuals feel they have failed in their social obligations (Kotera et al., 2023a; 2023b). Workplace policies may formally allow leave, but cultural expectations encourage rapid return, even when clinically harmful (Kotera et al., 2022). Families may hesitate to seek support out of concern that doing so signals inadequate contribution to group harmony (Kotera et al., 2023a, 2023b). None of these dynamics stems from the rejection of human rights. Rather, they reflect a moral economy where value is tied to fulfilling expected roles and demonstrating effort.

Analogous “earned worth” logics can emerge in societies shaped by different moral traditions (e.g., Confucian, Protestant, socialist or strong family duty norms). However, these logics are not culturally identical: they differ in the specific virtues emphasised (e.g., harmony vs. individual responsibility), the social arenas where judgement is applied (family, workplace and state) and how stigma and deservingness are expressed. The point is, therefore, not that the phenomenon is universal, but that similar forms of contribution-based deservingness can take distinct cultural shapes, supporting the need for culturally engaged implementation.

## The clash with rights-based mental health

Table 1 summarises common points of ethical dissonance: how rights-based principles may be interpreted within contribution-based moral economies. These contrasts are not incompatibilities in principle; they highlight where the moral meaning of support, rest and entitlement can diverge and thereby shape help-seeking and implementation.

**Table 1.** Where rights-based reforms can be morally misread in contribution-based contexts Table 1. long description.

Rights-based principles/values	Contrasts within contribution-based moral economies (potential moral misreadings)
Worth is unconditional	Worth is earned through social and economic contribution
Support is a right, not a reward	Support is legitimate only when a contribution has been demonstrated
Rest and recovery do not diminish one’s value	Rest may be interpreted as indulgence or irresponsibility
Autonomy and dignity persist regardless of functional capacity	Non-contribution generates moral judgement and shame

Thus, rights-based frameworks do not simply introduce new services: they introduce new moral expectations. When they enter cultural contexts where worth is contribution-based, they may be experienced as ethically dissonant. People may avoid seeking help not because they reject rights, but because they fear violating cultural norms about what makes a person worthy (Yang et al., 2014). In this account, the moral economy of worth is not a “barrier” separate from stigma, but part of the cultural substrate that shapes what stigma is about locally. Where worth is tightly coupled to contribution and self-discipline, mental distress (especially when not visibly embodied) can be moralised as insufficient effort or failure of responsibility, generating public stigma and self-stigma (Link and Phelan, 2001; Corrigan and Watson, 2002; Kotera et al., 2023a; 2023b). Stigma is therefore treated here as the more proximal mechanism linking moral economies to help-seeking, engagement and implementation. Mental health practitioners may feel tension between professional values and community moralities (Kleinman and Benson, 2006; Hatzenbuehler, 2016). Policymakers may frame support programmes in ways that inadvertently activate stigma.

This is not a marginal issue. In global mental health, culturally grounded moral economies are often treated as “barriers” to be removed, rather than as coherent systems of meaning that organise shame, responsibility and help-seeking. When reforms overlook these moral logics, they may misread reluctance as resistance, design services that feel morally risky to use and inadvertently intensify stigma, thereby undermining uptake and impact. Of course, implementation of rights-based services is also constrained by non-cultural barriers, such as resource and workforce limitations, infrastructure gaps, organisational resistance and political will, so the cultural lens proposed here should be understood as complementary rather than exhaustive.

## Integrating moral economies into rights-based mental health

A culturally viable rights-based approach does not treat contribution-based moralities as simply “wrong” or to be replaced. Instead, it engages them as moral grammars that organise legitimacy and shame, and it seeks to reduce the moral risk attached to help-seeking while keeping rights-based commitments explicit.

“Working with” an existing moral economy does not mean leaving it unchanged; it means expanding and reinterpreting it from within (e.g., broadening what counts as contribution) rather than replacing it with an external moral vocabulary. A key risk of this approach is that it could be misread as endorsing conditional deservingness – that is, that people must prove worth to merit care. The strategies below are therefore framed as implementation supports, not as new eligibility criteria, and they retain the normative claim that dignity and access to care are unconditional.

### Broaden what counts as a contribution

In contribution-based moralities, worth is tied to usefulness and responsibility. Rights-based services gain legitimacy when societies recognise that contribution is not only paid labour. Caregiving, community participation, relational maintenance and self-management (e.g., sleep, treatment adherence and relapse prevention) (Merlo and Fagundes, 2025) can be positioned as socially valuable work, often essential for group stability.

Example: Services can explicitly recognise “contribution” as self-management and relational work (e.g., attending appointments, practising sleep routines, relapse prevention, caregiving), and reflect this in care plans and recovery reviews.

### Reframe rest and recovery as duty-consistent

Rest is vulnerable to being interpreted as indulgence (Kotera, 2021). Reframe it as responsible stewardship of one’s functional capacity: recovery is not withdrawal from duty, but maintenance and restoration of the ability to contribute. This framing resonates especially in long-term oriented contexts, where short-term sacrifice is acceptable when it protects future obligations.

Example: Sick-leave letters and clinician conversations can frame rest as “stewardship of future participation” (e.g., “taking time now helps you return safely and reduces burden on family/work later”).

### Design policy language and eligibility rules to avoid moral “red flags”

Some terms and criteria inadvertently activate suspicions of undeserved reward (e.g., language that frames support as discretionary “help” or “approval,” or that defines eligibility primarily through incapacity and deficit labels rather than participation and inclusion). Rights-based guidance already points in this direction by recommending accessible, non-technical language and permitting non-medical/culturally meaningful descriptions of distress (e.g., “emotional distress” and “unusual experiences”), rather than relying on narrow clinical labels (World Health Organization, 2025). In the same spirit, national guidance has explicitly avoided traditional terms such as “serious mental illness” to respect multiple understandings of distress (NHS England, 2025).

This extends existing rights-based guidance by highlighting that, in contribution-based moral economies, welfare-eligibility language can be heard as indulgence or unearned reward even when the intent is rights protection. Practically, eligibility rules can therefore be framed around maintaining inclusion and participation (consistent with CRPD Article 19’s emphasis on community living and participation) rather than around deficit formulations (“because you cannot”) (United Nations, 2006). For example, the same eligibility decision can be communicated in two ways: Deficit framing: “Support is available because your condition prevents you from working or fulfilling expected roles.” Participation framing: “Support is available to help you remain included and participate in community, family, and work life, with adjustments as needed.”

Example: Phrasing that emphasises incapacity (“because you cannot…”) can inadvertently cue shame and “undeservedness,” whereas participation-oriented phrasing can reduce moral threat.

### Use culturally credible narratives to renegotiate norms

Abstract rights language often fails to shift moral intuitions. Lived-experience accounts, especially from respected in-group members, can model a new moral interpretation (Braddock and Dillard, 2016): seeking help is responsible, prevents burden on others and protects relationships (Thornicroft et al., 2016). Narratives can also separate “rest” from “selfishness,” and “support” from “moral failure.” (Slade et al., 2024).

Example: Short peer narratives can model “help-seeking as responsible” (e.g., “getting support early helped me keep my commitments to my family/work”).

### Engage institutions that shape moral judgement

Moral economies are produced and reinforced through everyday institutions (families, workplaces, schools and faith/community organisations), but they are also shaped by wider structures, including welfare regimes, labour markets, media/NGOs and transnational norms, which can stabilise or contest local expectations of worth and responsibility (Wheeler, 2019). Importantly, these moral economies are not only “cultural”; they are also sustained by political-economic conditions. Labour-market insecurity, welfare conditionality and productivity-oriented norms (amplified through economic globalisation) can intensify the coupling of worth with paid work and measurable contribution, even within the same national culture. A cultural-implementation lens can complement political-economy analysis by showing how structural pressures are interpreted through locally salient moral logics in everyday life and services. Sustainable change requires multilevel moral dialogue: co-developing messaging with community institutions, training supervisors and frontline staff and aligning programme design with participation-oriented goals so that help-seeking is legitimised while valued commitments to responsibility and harmony are preserved.

Example: Partner with employers/schools/faith groups to co-produce messages that legitimise help-seeking as responsible maintenance, and train supervisors to avoid “effort-blame” framing.

## Conclusion

Human dignity may be universal, but the cultural foundations of who is perceived as deserving support are not. Moral economies of worth (shaped by restraint, collectivism and long-term orientation) profoundly influence how rights-based mental health reforms are understood and accepted. If global mental health is to be truly global, it must expand its conceptual frameworks to include these cultural moralities, not merely as contextual barriers but as central determinants of meaning, legitimacy and care.

Integrating rights-based approaches with moral economies of contribution does not weaken human rights. It has the potential to strengthen their cultural resonance, ethical grounding and practical viability. Only by engaging these moral systems can rights-based mental health become sustainable and globally meaningful.

## Data Availability

This study did not involve the novel creation, generation, or analysis of primary research materials. All relevant literature is cited in the References.

## Long descriptions

### Table 1. Long description

The table consists of two columns and four rows of comparative data.

* Row 1: Rights-based principle states worth is unconditional. The contribution-based contrast is that worth is earned through social and economic contribution.

* Row 2: Rights-based principle states support is a right, not a reward. The contribution-based contrast is that support is legitimate only when a contribution has been demonstrated.

* Row 3: Rights-based principle states rest and recovery do not diminish one’s value. The contribution-based contrast is that rest may be interpreted as indulgence or irresponsibility.

* Row 4: Rights-based principle states autonomy and dignity persist regardless of functional capacity. The contribution-based contrast is that non-contribution generates moral judgement and shame.

Navigate back to Table 1..
