# Supplementary material for: The moral economy of worth: Why rights-based mental health must engage cultural logics of contribution
Source: Glob Ment Health (Camb). 2026 Jun 16;13:e150. doi: 10.1017/gmh.2026.10255 (PMC13373263; doi:10.1017/gmh.2026.10255)
Supplement: Peer review history

# Reviewer report: Review: The moral economy of worth: Why rights-based mental health must engage cultural logics of contribution — R0/PR2

**DOI:** 10.1017/gmh.2026.10255.pr2

**Authors:** Laurence Kirmayer

**Affiliations:** Psychiatry, McGill University, Canada

This is a clear, well-written and useful paper. I have only to minor suggestions:

(1) Both the ganbaru and amae value systems are presented without much explication. Amae, in particular, has been described as something tolerated and accepted, so a few words about the nature of the ambivalence around it would be useful.

(2) There fact that there are many ethical systems that emphasize “earned worth” (Confucian, Protestant, socialist, etc.) suggests that universal human rights are not simply at odds with specific cultural values but represent an intervention to assert a set of values or ideals that aim to have a transformative effect on culture. Is there a risk that by operating within the moral economy of having to prove one’s worth to obtain care, we undermine this call for change?

# Reviewer report: Review: The moral economy of worth: Why rights-based mental health must engage cultural logics of contribution — R0/PR3

**DOI:** 10.1017/gmh.2026.10255.pr3

I. General Assessment

Thank you for the opportunity to review this manuscript. This Perspective addresses a genuine and important topic: the gap between the universalistic values underlying rights-based mental health approaches and the culturally embedded moral logics that can shape understanding of worth, deservingness, and support. The central thesis, that contribution-based moralities could create ethical dissonance undermining the uptake of rights-based reforms, is plausible and policy-relevant. The paper has real potential to contribute to the global mental health literature.

Several strengths deserve to be acknowledged. The paper is grounded in a rich and well-selected literature, drawing productively across cross-cultural psychology, mental health policy, and implementation research. The introduction is clear and well situated in the international context, and the rhetorical positioning of the paper as strengthening rather than challenging the rights-based agenda is sound. The section on cultural configurations is clearly written and succeeds in illustrating how cultural dimensions can account for variation in social representations of individual worth. The Japanese case is effectively used to demonstrate how these dynamics translate into concrete health behaviours and clinical experience. The clash section is useful and concrete in mapping tensions between rights-based principles and contribution-based moral economies. Finally, the strategies section provides the paper’s clearest policy value, and the core framing, working with existing moral economies rather than against them, is a conceptually original and practically promising contribution.

Nonetheless, major revisions are required to address issues affecting the argumentative coherence, epistemological positioning, and practical operationalisation of the article. Cross-cutting remarks are first described, followed by section-specific comments.

II. Cross-Cutting Remarks

Comment n°1: Across the manuscript are several cases where it is not clearly differentiated whether the author refers to empirical findings, theoretical propositions, or speculative arguments. Several claims that are advanced as established facts are better characterised as plausible hypotheses. Representative examples are discussed in the section-by-section comments below (comments n°8, 9, 12, 16, 22), but the authors are invited to review the entire manuscript to further clarify the distinction between what is known, what is proposed and what is conjectured.

Comment n°2: The concept of “moral economy” is central to the entire argument but is neither defined nor anchored in the literature. Is it borrowed as an established analytical concept, or used metaphorically? This ambiguity weakens the theoretical foundation of the paper and should be resolved at the outset. Clarifying this will improve the understanding of the proposed “moral economy of worth”.

Comment n°3: An important angle is not addressed in the article: to what extent is the formulation of fundamental human rights itself shaped by a Western system of values (e.g., Mutua, 2002)? If the framework that is being promoted globally is already culturally situated, the dissonance described in this paper is not simply a clash between universal rights and local moralities, but between competing cultural frameworks. This debate exists in the critical global mental health literature (e.g., Summerfield, 2012) and deserves acknowledgement. It would actually strengthen the paper’s argument for cultural engagement.

Mutua, M. (2002). Human rights: A political and cultural critique. University of Pennsylvania Press.

Summerfield D. Afterword: Against “global mental health”. Transcultural Psychiatry. 2012;49(3-4):519-530. doi:10.1177/1363461512454701

Comment n°4: The paper consistently frames contribution-based moralities as cultural phenomena, but does not engage with the structural and economic conditions that produce and sustain them. The role of capitalist ideology and economic globalisation in shaping the perception that individual worth is tied to productive contribution is a significant omission. Presenting contribution-based worth as an essentially cultural characteristic, without acknowledging its entanglement with global economic structures, risks essentialising what is also a product of political economy. The authors are invited to acknowledge this dimension, even briefly, and to consider whether it strengthens rather than undermines their case for cultural engagement.

Comment n°5: Across the paper, the authors frame contribution-based cultural logics as barriers to rights-based approaches implementation. A question arises regarding the level of analysis at which the argument operates: the mechanism through which they interpret the effects of these cultural logics on help-seeking and clinical experience is, consistently, stigma and self-stigma. Could they rather be describing a culturally specific dimension of stigma centred on social worth and perceived contribution? If this reading is correct, cultural moral economies may be better understood as the substrate that gives stigma its specific local content, rather than as a primary barrier in themselves, with stigma remaining the more proximal mechanism linking cultural context to health behaviour. The authors are invited to reflect on this distinction and to clarify the relationship between moral economy and stigma as levels of analysis. Engaging more explicitly with the stigma literature could considerably strengthen the paper’s theoretical contribution.

III. Section-by-Section Comments

1. Introduction

Comment n°6: The rights-based approach is introduced without sufficient contextualisation. What does this framework concretely entail for mental health care (e.g., in terms of access, continuity, quality of services)? Spelling this out would help readers immediately grasp what is at stake when cultural moral logics create friction with this approach.

Comment n°7: Relatedly, the authors should clarify what the ethical assumption of inherent human worth is operationally supposed to produce in mental health systems (e.g., reducing inequalities in care access). Without this, the downstream consequences of cultural misalignment remain abstract.

Comment n°8: Line 106 authors write: “Global mental health has largely overlooked these cultural moral systems”. This claim is the argumentative pivot of the entire paper, yet it is asserted without citation or exemplification. This is the point that most needs to be substantiated: which specific frameworks, policy documents, or dominant approaches are being characterised here, and on what evidence?

Comment n°9: Lines 106–109, authors write: “As a result, rights-based approaches often confront silent but powerful friction: not because communities reject rights, but because they operate within alternative moral frameworks that structure the meaning of value, responsibility, and entitlement. ». This is presented as an established fact, but is rather a plausible and interesting hypothesis. The sentence should therefore be reformulated in the conditional.

Comment n°10: The authors could strengthen their argument by acknowledging that rights-based approaches implementation additionally faces barriers that are, if not at all, indirectly related to cultural moral logics (e.g., lack of resources and infrastructures, institutional and political will, resistance to organisational change). Recognising the diversity of these obstacles would help situate the specific overarching contribution of the cultural lens proposed here, rather than leaving the impression that moral economy is the primary or sole obstacle.

2. Cultural Configurations Behind Contribution-Based Worth

Comment n°11: The use of Hofstede’s model is the most significant methodological concern in this section. The model is mobilised as though its cross-cultural validity were uncontested, whereas it has been subject to sustained and substantial criticism in the literature (e.g., McSweeney, 2002). The authors should clarify why the Hofstede framework remains a useful heuristic for their purposes despite these limitations.

McSweeney, B. (2002). Hofstede’s model of national cultural differences and their consequences: A triumph of faith-a failure of analysis. Human relations, 55(1), 89-118.

Comment n°12: The authors should clarify the epistemological status of this section at the outset. Are the three dimensions proposed (Restraint, Collectivism, Long-Term Orientation) a theoretical proposition from the authors, i.e., a framework they are putting forward, or a synthesis of well-established empirical findings? The answer shapes how the section should be read and evaluated.

Comment n°13: Lines 129–135, the authors claim that collectivist social logics produce resistance to receiving support without contributing. However, collectivist social contracts may equally imply an obligation to care for vulnerable members of the group. It is possible that contribution-based moralities create stigma and self-stigma without necessarily negatively impacting care per se (e.g., reducing access)? For example, individuals may receive care while simultaneously experiencing shame and internalised negative worth. This directly relates to comments n°6 and 7 above.

3. Japan as an Illustrative, Not Exceptional, Case

Comment n°14: Lines 166–168 introduce a claim that is in tension with the paper’s overarching argument: “Similar patterns appear across societies shaped by Confucian ethics, Protestant work ethics, socialist labour ideology, or strong family duty norms. What appears culturally specific is often structurally similar once we examine the underlying moral logic.” If the underlying moral logic is structurally similar across Confucian, Protestant, socialist, and family duty-based societies, what remains distinctively cultural about the phenomenon the authors are describing? The authors should clarify whether they are advancing a culturalist thesis or a thesis about universal moral logics that express themselves differently across contexts. This distinction has significant implications for the paper’s argument and for the strategies proposed.

4. The Clash with Rights-Based Mental Health

Comment n°15: Table 1 would benefit from clearer column labelling. As currently titled, the distinction between what the left column represents (aspirational principles) and what the right column represents (potential moral misreading in specific cultural contexts) is not immediately apparent. Suggested revision: “Rights-based principles/values” and “Contrasts within contribution-based moral economies.”

Comment n°16: Lines 179–181: “When they enter cultural contexts where worth is contribution-based, they create ethical dissonance” should be reformulated in the conditional, consistent with the general concern about epistemic marking raised above.

5. Integrating Moral Economies into Rights-Based Mental Health

Comment n°17: The five strategies are formulated at a level of generality that limits their operational usefulness, especially for a paper oriented toward implementation. The authors could illustrate each strategy with at least one concrete example drawn from existing programmes or documented experiences. This would make the contribution considerably more actionable for practitioners and policymakers.

Comment n°18: An internal tension in the proposed strategies should be acknowledged. Strategies 1 (broadening what counts as contribution) and 4 (using culturally credible narratives to renegotiate norms) both involve modifying the existing moral system, not merely adapting to it. This directly contradicts the principle of “working with the existing moral economy” stated at the outset of the section. The authors should explicitly address this. Perhaps the distinction is that these strategies seek to expand the moral economy from within, rather than replacing it: if so, this should be stated clearly.

Comment n°19: The framing at lines 194–197 “A culturally viable rights-based approach does not treat contribution-based moralities as simply “wrong” or to be replaced. Instead, it works with the existing moral economy, shifting what counts as contribution, and reframing support so that rights feel morally legitimate rather than morally risky” is conceptually sound and deserves more argumentative weight than it currently receives.

Comment n°20: Line 214: “Some terms and criteria inadvertently activate suspicions of undeserved reward”: an example or two would considerably clarify this claim.

Comment n°21: Lines 224–227: The recommendation to frame eligibility rules around participation rather than deficit formulations is interesting. A hypothetical example contrasting the two types of formulation would make this recommendation immediately usable.

6. Conclusion

Comment n°22: One point requires attention: the claim that the proposed approach “strengthens the cultural resonance, ethical grounding and practical viability” of human rights is too assertive. Suggestion is to exercise greater caution: “It has the potential to strengthen…” .

IV. Recommendation

Major revision. The paper addresses a genuine and important problem, and the central thesis has real potential to contribute to the global mental health literature. However, it requires substantial work on three fronts: conceptual clarification (particularly the definition and positioning of the moral economy framework, and the limits of the Hofstede model), epistemic rigour (consistent marking of the status of claims throughout the manuscript), and operationalisation of the proposed strategies.

I look forward to reviewing the revised manuscript and remain available should the authors require clarification on any of the points raised above.

# Editor report: Recommendation: The moral economy of worth: Why rights-based mental health must engage cultural logics of contribution — R0/PR4

**DOI:** 10.1017/gmh.2026.10255.pr4

**Affiliations:** Director, National Institute of Mental Health, Czech Republic

No accompanying comment.

# Editor report: Decision: The moral economy of worth: Why rights-based mental health must engage cultural logics of contribution — R0/PR5

**DOI:** 10.1017/gmh.2026.10255.pr5

**Affiliations:** Johns Hopkins University Bloomberg School of Public Health, United States

No accompanying comment.

# Reviewer report: Review: The moral economy of worth: Why rights-based mental health must engage cultural logics of contribution — R1/PR7

**DOI:** 10.1017/gmh.2026.10255.pr7

**Authors:** Laurence Kirmayer

**Affiliations:** Psychiatry, McGill University, Canada

The author’s have responded well to the earlier reviews and the article makes a very important contribution. Their arguments are conssitent with in an earlier review (Kirmayer, L. J. (2012). Culture and context in human rights.In: udley, M., Silove, D., & Gale, F. (Eds.), Mental health and human rights: Vision, praxis and courage (pp. 95-112) Oxford Univeristy Press) but they have developed very useful suggestions for advanced rights-based mental health through close attention to culture.

# Reviewer report: Review: The moral economy of worth: Why rights-based mental health must engage cultural logics of contribution — R1/PR8

**DOI:** 10.1017/gmh.2026.10255.pr8

I thank the authors for having carefully addressed each of the points raised in the initial review. The responses are comprehensive, and demonstrate genuine engagement with the critiques formulated. The revisions brought to the manuscript, in particular the clarification of the moral economy framework, the more nuanced epistemic marking of claims throughout the text, the acknowledgement of the limitations of the Hofstede model, and the enrichment of the proposed strategies with concrete examples, substantially strengthen both the argumentative coherence and the practical value of the paper. The revised manuscript is considerably clearer in its theoretical positioning and more rigorous in its handling of the evidence. The concerns raised have been adequately addressed. My recommendation is to accept the manuscript for publication.

# Editor report: Recommendation: The moral economy of worth: Why rights-based mental health must engage cultural logics of contribution — R1/PR9

**DOI:** 10.1017/gmh.2026.10255.pr9

**Affiliations:** Director, National Institute of Mental Health, Czech Republic

Thank you for this valuable contribution to the global mental health discussion.

# Editor report: Decision: The moral economy of worth: Why rights-based mental health must engage cultural logics of contribution — R1/PR10

**DOI:** 10.1017/gmh.2026.10255.pr10

**Affiliations:** Johns Hopkins University Bloomberg School of Public Health, United States

No accompanying comment.
